# ANATOMICAL VARIATIONS OF THE CELIAC TRUNK: A SYSTEMATIC REVIEW

**DOI:** 10.1590/0102-672020180001e1403

**Published:** 2018-12-06

**Authors:** Priscele Viana dos SANTOS, Ana Beatriz Marques BARBOSA, Vanessa Apolonio TARGINO, Nathalie de Almeida SILVA, Yanka Costa de Melo SILVA, Felippe BARBOSA, André de Sá Braga OLIVEIRA, Thiago de Oliveira ASSIS

**Affiliations:** 1 University Center UNIFACISA), João Pessoa, PB; 2 Department of Morphology of the Federal University of Paraíba Campina Grande-PB), Brazil.

**Keywords:** Abdomen, Abdominal aorta, Celiac trunk, Anatomical variation, Abdome, Aorta abdominal, Tronco Celíaco, Variação anatômica

## Abstract

**Introduction:**

The celiac trunk (CT) is one of the abdominal portion branches of the aortic artery and, together with the superior mesenteric and inferior mesenteric arteries, participates in the abdominal viscera vascularization through a series of anastomoses. Absence of CT or variation in the number of terminal branches implies in varied abdominal arteries origins, which may have implication in surgical approaches.

**Objective:**

To analyze the anatomical variations of the celiac trunk and possible associated surgical clinical implications.

**Methods:**

It is a systematic review of articles indexed in the PubMed, Lilacs, SciELO, Springerlink, Scienc Direct and Latindex databases from August to September 2017. Original articles involving the anatomical variations of the celiac trunk in humans were included. The presence/absence of the celiac trunk, the number of terminal branches and the place of origin of its branches in variant cases of the normal anatomical pattern, were considered for this study.

**Results:**

At the end of the research, 12 articles were selected, characterized by sample, anatomical structure evaluation method and main results. The normal anatomical pattern was the most prevalent in most studies (75.0%). CT was absent in 41.7% of the findings. The most prevalent anatomical variation was the presence of CT with bifurcation (66.7%). It was also observed the origin of the common and splenic hepatic arteries from the mesenteric arteries (25.0%). The presence of only one branch (16.7%) and quadrifurcation (8.33%) were other findings.

**Conclusion:**

CT variations are not uncommon findings, with different anatomic variants being reported. Thus, the importance of knowing the possible variations of this structure is emphasized, which may have implications for surgical interventions and imaging studies related to the abdominal region.

## RESUMO

**Introdução:**

O tronco celíaco (TC) surge da aorta abdominal e juntamente com as artérias mesentérica superior e mesentérica inferior participa da vascularização de vísceras abdominais por meio de uma série de anastomoses. Ausência do TC ou variação no número de ramos terminais implica em origens variadas das artérias abdominais, o que pode ter implicação em abordagens cirúrgicas.

**Objetivo:**

Analisar as variações anatômicas do TC e as possíveis implicações clínico/cirúrgicas associadas.

**Métodos:**

Trata-se de uma revisão sistemática de artigos indexados nas bases de dados PubMed, Lilacs, SciELO, Springerlink, Scienc Direct e Latindex, no período de agosto a setembro de 2017. Foram incluídos artigos originais envolvendo as variações anatômicas do TC em humanos. Considerou-se para este estudo a presença/ausência do TC, o número de ramos terminais e o local de origem de seus ramos em casos variantes do padrão anatômico normal. A coleta foi realizada por dois revisores independentes.

**Resultados:**

Ao final da busca foram selecionados 12 artigos, caracterizados quanto à amostra, método para avaliar a estrutura anatômica e principais resultados. O padrão anatômico normal foi o mais prevalente na maioria dos trabalhos (75,0%). O TC foi ausente em 41,7% dos achados. A variação anatômica mais prevalente foi a presença do TC com bifurcação (66,7%). Observou-se, ainda, a origem das artérias hepática comum e esplênica a partir das artérias mesentéricas (25,0%). A presença de apenas um ramo (16,7%) e quadrifurcação (8,33%) foram outros achados presentes.

**Conclusão:**

Variações do TC não são achados incomuns, sendo relatados diferentes variantes anatômicas. Desse modo, ressalta-se a importância sobre o conhecimento das possíveis variações dessa estrutura, o que pode ter implicação em intervenções cirúrgicas e exames de imagem relacionados à região abdominal.

## INTRODUCTION

The celiac trunk (CT) arises from the abdominal part of the aortic artery, and in its normal pattern it is possible to verify the existence of three terminal branches, the left gastric artery, which runs through the smaller curvature of the stomach, the splenic artery, which follows tortuous by the posterior superior margin of the pancreas to the spleen, and the common hepatic artery, which divides into gastroduodenal for the pancreas and duodenum vascularization, and its own hepatic artery, which will supply the liver. This trifurcation is the normal pattern of TC present in about 89% of individuals regardless of gender, whereas anatomic variations of the bifurcation type occur in 11% of the population, and their absence is rare, affecting 0.2% of the individuals². 

During the abdomen developing process, the primitive arteries form three arteries related to the digestive system viscera that correspond to the celiac trunk, superior mesenteric artery and inferior mesenteric artery. The descendent longitudinal anastomoses at the front of the aorta forms the omphalomesenteric artery, the anterior longitudinal anastomoses, during the embryological development, namely between the future celiac trunk and the superior mesenteric artery, giving rise to the embryological development of the arterial hepatic trunk. In the absence of celiac trunk, the descending and anterior longitudinal anastomoses regress completely; however, the roots of the ventral segmental arteries do not. The 10^th^ primitive root of the ventral segmental artery becomes the left gastric artery; the 11^th^ becomes the splenic artery; the 12^th^ becomes the common hepatic artery^7.^ The absence of the celiac trunk is a rare anomaly with incidence rates varying from 0.1 to 2.6%^27, 28.^ Only 31 cases of missing celiac trunk were reported worldwide and about 1/3 of these cases were detected by imaging studies, while other variations were observed during anatomical dissections^13^.

According to Gluck, Gerhardt, Schoroder^10^ knowledge of celiac trunk variations is important for surgeons during hepatic transplantation, laparoscopic surgery, radiological interventions as well as penetrating lesions in the abdomen. In addition, knowledge of unique variations of celiac trunk absence may be useful in planning and performing radiologic interventions such as celiac and chemoembolization of liver tumors^1.^ Changes in the celiac artery may increase both the difficulty and the risk of radical gastrectomy^13^. 

This information motivated this study, that has as objective to demonstrate the anatomical variations of the celiac trunk and its clinical implications in the human.

## METHOD

This is a systematic review. The electronic search was performed from August to September 2017. For the accomplishment of this study, the following databases were consulted: SciELO (Scientific Electronic Library Online); PubMed (National Library of Medicine and National Institute of Health); Science Direct; Springerlink; and Latindex (Regional Online Information System for Scientific Journals of Latin America). We selected articles without time restriction, in English and Portuguese. For the prospection of the studies, the descriptors were used in combination with boolean operators (AND). In SciELO the combination was: “Celiac trunk”, AND “anatomical variation”. In PubMed, Science Direct, Springerlink and Latindex: “Human celiac trunk” AND “anatomical variation”.

For the calculation of the number of studies, it was verified if they were not repeated on more than one basis, each article being considered only once. From the identified studies, those that fulfilled the criteria for inclusion were selected considering the titles and abstracts. Original articles involving the anatomical variations of the celiac trunk in humans, prioritizing studies of greater relevance were included in this review. We excluded review articles, and studies with models involving animals.

The articles were critically analyzed through an interpretation guide, used to evaluate their individual quality, based on the studies^11^ and adapted by Mcdermid et al.^17^. The articles quality evaluation items are expressed by scores in Table 1, in which 0=absent; 1=incomplete; and 2=complete.

**TABLE 1:** Characteristics of the studies that evaluated the relation of the anatomical variations of the celiac trunk in humans

Study (year)	Sample	Method	Main results
Araujo Neto SA et al. ^2^	60 patients	Computedtomography	In 90% of the cases there was no change in CT, 8.3% of the patients had hepatosplenic trunk and 1.7% had the hepatogastric trunk.
Clement MI et al. ^6^	43 adults corpses e 596 exams	Dissection and angiographic exams	The results were divided into celiac trunk type I (complete) in 90.5% of the sample and celiac trunk type II (incomplete) in 9.5% of the sample. In type I, the trunk was bifurcated, trifurcated or quadfurcated, in the latter, with accessory branch. Those of the incomplete type presented hepatosplenic or gastroesplenic divisions.
Fahmy D Sadek H ^9^	Single	Computerdtomography	Absence of celiac trunk. The gastric, splenic, hepatic and mesenteric arteries arose independently of the abdominal aorta.
Chen H et al. ^5^	974 corpses	Corpse dissection	In 89.8% of the cases, classical trifurcation of the celiac trunk was observed. A common hepatosplenic trunk and a gastrohepatic trunk were observed in 4.4%. A common hepatic artery resulting from the superior mesenteric artery or directly from the aorta was present in 3.5%. A hepatosplenomesenteric trunk and a celio-mesenteric trunk were found in 0.7%.
Prakash et al. ^19^	50 corpses	Corpse dissection	The gastric, hepatic and splenic arteries appeared from the celiac trunk in 86% of the corpses. In 76% the origin of the gastric artery was proximal to the bifurcation of the celiac trunk, in the hepatic and common splenic artery. In one case, the three branches emerged directly from the abdominal aorta.
Badagabettu SN et al. ^4^	Single	Corpse dissection	Absence of celiac trunk. Common hepatic artery, left gastric and splenic artery with independent origin in the aorta. Trifurcation of the common hepatic artery in the right hepatic, left hepatic and gastroduodenal arteries.
Petrella S et al. ^18^	89 corpses	Corpse dissection	In 67.90% of the sample, the left gastric artery was verified as the first branch of the celiac trunk, the splenic artery in 7.41% and in 22.22% in the three arteries forming the Haller tripod. It was observed as the last branch of the celiac trunk, the common hepatic artery in 19.12%, the splenic in 5.88%. In 82.02% the celiac trunk emitted the gastric, splenic and hepatic arteries, in addition to the three arteries, the celiac trunk emitted a gastroduodenal artery in 6.74%. The gastrosplenic trunk was observed in 3.37%, the common hepatic as the sole branch in 1.12% and in 1.12% absence of the celiac trunk.
Silveira LA et al. ^22^	21 corpses	Corpse dissection	Of the 21 cadavers, 6 (28.57%) presented anatomical variations of at least one of the branches of the celiac trunk, being the absence of the hepatic artery itself, the middle colic artery originating in the celiac trunk, the left gastric artery having origin in the abdominal aorta, the right hepatic artery originating in the superior mesenteric artery and two trunks that emerged from the abdominal aorta, a gastroesplenic and another hepatomesenteric.
Sehgal G et al.^21^	50 patients	Computed tomography	A variation in the origin site of the celiac trunk was observed in 55% of the cases. In 45.83%, the celiac trunk originated from the junction of T12-L1, in 29.17% it originated in front of the T12 vertebra, 22.92% in front of the L1 vertebra and at the junction of T11-T12 2, 08%. The length varied between 6 mm and 22 mm, and the dimensions of the trunk ranged from 4 mm to 10 mm.
Sztika D et al. ^25^	Single	Corpse dissection	There was an incomplete celiac trunk, the hepatosplenic trunk from which the common hepatic artery and the splenic artery arise. The left gastric artery appears separately 0.5 cm from the origin of the celiac trunk, directly from the abdominal aorta.
Ugurel MS et al. ^26^	100 patients	Multidetector Angiography	There was a trifurcation in the celiac trunk in 89% and bifurcation in 8% of the cases. The celiac trunk was absent in 1%, a hepatosplenomesenteric trunk was observed in 1% and a splenomasenteric trunk was present in 1%.
Zagyapan R et al. ^29^	152 patients	Digital subtraction angiography	There was a classic trifurcation of the celiac trunk in 62.5% of the patients. The variant right hepatic artery arose from the superior mesenteric artery in 17.8%. The hepatic artery as branch of the left gastric artery in 13.1%. The common hepatic artery resulting from the superior mesenteric branch was observed in 6.6% of the patients.

### Statistical analysis

The search was performed by two independent reviewers, and the interobserver agreement analysis was performed using the Kappa test using the Bioestat V 5.0 software, according to Landis and Koch^15^ method. The value found was K=0.78 (Substantial agreement).

## RESULTS

**FIGURE 1:**
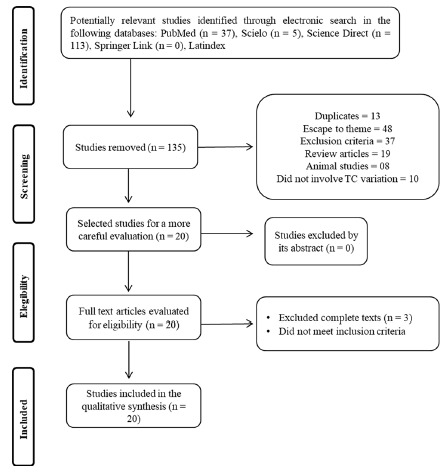
Search and selection of the studies for the systematic review according to the PRISMA recommendations

A summary of the electronic search in the databases and the respective directions for inclusion are presented in Figure 1. Initially, 155 articles were identified, of which 135 were excluded because they did not have relevant data or because they were in duplicates, remaining 20, which were submitted to analysis of titles and abstracts and verification of inclusion and exclusion criteria. Of these, 20 were read in full, of which only 12 articles adequately fulfilled all inclusion criteria and were thus selected for analysis ^2, 4-6, 9, 18, 19, 21, 22, 25, 26, 29.^

Table 1 shows the main findings of the studies used for discussion. It is stratified by year of publication, sample, method used and main results.

The descriptive and critical analyzes of the studies were carried out in a qualitative and quantitative way, based on the analysis of the nine domains of the AHRQ scale, which were in the 54-86 score range.

**FIGURE 2:**
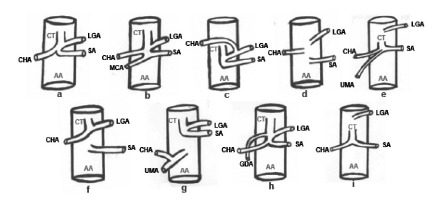
Normal celiac trunk (a) and its variant forms reported in the studies (b, c, d, e, f, g, h, i). AA=aorta artery; CT=celiac trunk; CHA=common hepatic artery; LGA=left gastric artery; SA=splenic artery; MCA=middle colonic artery; UMA=upper mesenteric artery, GDA=gastroduodenal artery.

The variant forms of the celiac trunk, found in the analysis of the selected works, totaled eight forms and were represented in Figure 2 for a better understanding. The central vascular axis represents the abdominal segment of the aortic artery.

**TABLE 2:** Analyses of the quality of the articles evaluating the anatomical variations of the celiac trunk in humans

STUDIES	EVALUATION CRITERIA												
	1	2	3	4	5	6	7	8	9	10	11	12	Total (%)
Petrella *et al.* (2007)	2	0	1	2	2	NA	2	2	2	2	0	1	72.72
Huayue *et al.* (2009)	2	0	2	2	2	NA	2	2	2	2	0	1	77.27
Silveira *et al.* (2009)	2	2	1	2	1	NA	2	1	2	2	0	2	77.27
Ugurel *et al.* (2010)	2	0	1	2	2	NA	2	1	2	2	0	0	63.63
Sztika *et al.* (2011)	2	0	1	2	1	NA	2	2	2	2	0	1	68.18
Prakash *et al.* (2012)	1	0	1	1	2	NA	1	2	1	1	0	2	54.54
Sehgal, *et al.* (2013)	2	2	1	2	2	NA	2	2	2	2	0	2	86.36
Zagyapan *et al.* (2014)	2	0	1	2	2	1	2	1	2	2	0	2	70.83
Araujo *et al.* (2015)	2	2	1	2	2	NA	2	2	2	2	0	1	81.81
Fahmy e Sadek*.* (2015)	0	0	1	1	1	NA	2	1	0	1	0	0	31.81
Clement *et al.* (2016)	2	2	1	2	2	2	2	1	2	2	0	2	83.33
Badagabettu *et al.*(2016)	1	0	1	1	1	NA	2	2	1	1	0	2	54.54

Abbreviations: NA, not applicable* Evaluation criteria: 1. thorough review of the literature to define the research question; 2. specific inclusion/exclusion criteria; 3. specific assumptions; 4. appropriate scope of psychometric properties; 5. sample size; 6. follow-up; 7. the authors referred specific procedures for administration, punctuation and interpretation of procedures; 8. measurement techniques were standardized; 9. data were presented for each hypothesis; 10. appropriate statistics - timely estimates; 11. appropriate statistical error estimates; 12. valid conclusions and clinical recommendations.

## DISCUSSION

The present review sought to investigate the variant forms of celiac trunk that have been described from the analysis of cadavers and/or diagnostic imaging. Most of the studies included in this review (75.0%) showed that the normal anatomical pattern of celiac trunk division (item a, Figure 2) was the type of major occurrence. This is the standard expected for most individuals. During development, CT is the first ventral branch of the abdominal aorta, emerging at the T12 level. This trunk is divided into three terminal branches that, through a series of anastomoses, participate in the irrigation of abdominal viscera^9^. 

A study with corpses and imaging studies found that 90.5% of the sample presented the classic pattern of trifurcation^6^. Petrella et al.^18^ observed the trifurcation of the celiac trunk in left gastric, splenic and hepatic arteries common in 82.0% of the sample. In the study by Zagyapan, et al.^29^, the classic trifurcation of the celiac trunk occurred in 62.5% of the patients.

The absence of the celiac trunk was reported in five of the 12 studies of this review^4^ ^,^ ^9^ ^,^ ^18^ ^,^ ^19^ ^,^ ^26^. In these cases, the common gastric, splenic and hepatic arteries originated independently directly from the abdominal aorta (item d, Figure 2)^4^ ^,^ ^9^ ^,^ ^19^. During the developmental process, primitive arteries are longitudinally anastomosed formed and regress to some extent, where they remain throughout life. The absence of the celiac trunk occurs due to a complete regression of the anastomoses of the primitive arteries. However, the roots of the segmental arteries do not regress, and thus emerge directly from the abdominal aorta^9^.

The bifurcation of the celiac trunk, that is, the absence of one of its terminal branches, has been reported as the most common variant form of this structure, as observed in this review, in which 66.7% of studies presented this variation. When this variation was present, it was observed the formation of hepatosplenic, gastroesplenic and hepatograstric trunks^2^ ^,^ ^3^ ^,^ ^6^. About 11% of the general population presents this type of variation^2^. 

Through a survey with 60 patients using computerized tomography, 8.3% of the patients were found to have splenic hepatic trunk (with absence of left gastric art, Figure 2i), whereas 1.7% presented hepatogastric trunk (with absence of the splenic artery, Figure 2)^2^. For these variations, in gastrectomy cases, one should proceed with caution, since cases of left hepatic artery emerge from the left gastric artery, thus, with a section of the left artery, there may be a possible ischemia of the entire functional yellow liver lobe.

Other variations described indicate the origin of only one terminal branch (16.7%) or quadrifurcation of the CT scan (8.3%, Figure 2b or 2h). In the paper of Petrella et al.^18^, it was verified the formation of only one branch from the CT, this being the common hepatic artery. In the cases where four terminal branches were formed, an accessory branch was observed for irrigation of the abdominal structures^6^ and the origin of the gastroduodenal artery from the celiac trunk^18^, as demonstrated in the item h of Figure 2.

Variation was also observed regarding the order in the terminal branches origins of the celiac trunk. In a study with corpses, it was observed that, in most individuals, the first branch of the CT scan was the left gastric artery (67.9%). The splenic artery occurred in 7.4% of the cases and in 22.2% the three arteries trifurcated at the same height, forming the Haller’s tripod^18^. The left gastric artery is the first branch of the CT and runs cranially to the lesser curvature of the stomach, where it anastomoses with the right gastric artery^3^. 

Another finding in the study was the origin of one of the terminal branches of the celiac trunk from the mesenteric arteries. A research performed using angiographies observed that 1% of the patients presented a hepatosplenomesenteric trunk (Figure 2e) and 1% a splenomesenteric trunk^26^. Chen et al.^5^ report the presence of a hepatosplenomesenteric trunk as one of the anatomical variations observed in its sample. In this study we also observed the presence of a celiomesenteric trunk, denoting a common origin of celiac trunk and superior mesenteric artery.

## CONCLUSION

Celiac trunk variations are not uncommon findings, with different anatomic variants being reported. Thus, the importance of knowing the possible variations of this structure is emphasized, which may have implications for surgical interventions and imaging studies related to the abdominal region.
